# Understanding plastome evolution in Hemiparasitic Santalales: Complete chloroplast genomes of three species, *Dendrotrophe varians*, *Helixanthera parasitica*, and *Macrosolen cochinchinensis*

**DOI:** 10.1371/journal.pone.0200293

**Published:** 2018-07-05

**Authors:** Hye Woo Shin, Nam Sook Lee

**Affiliations:** 1 Interdisciplinary Program of EcoCreative, The Graduate School, Ewha Womans University, Seoul, Korea; 2 Department of Life Science, Ewha Womans University, Seoul, Korea; Chinese Academy of Medical Sciences and Peking Union Medical College, CHINA

## Abstract

Santalales is a large order, with over 2200 species, most of which are root or aerial (stem) hemiparasites. In this study, we report the newly assembled chloroplast genome of *Dendrotrophe varians* (140,666 bp) in the family Amphorogynaceae and the cp genomes of *Helixanthera parasitica* (124,881 bp) and *Macrosolen cochinchinensis* (122,986 bp), both in the family Loranthaceae. We compared the cp genomes of 11 Santalales including eight currently available cp genomes. Santalales cp genomes are slightly or not reduced in size (119–147 kb), similar to other hemiparasitic species, when compared with typical angiosperm cp genomes (120–170 kb). In a phylogeny examining gene content, the NADH dehydrogenase gene group is the only one among eight functional gene groups that lost complete functionally in all examined Santalales. This supports the idea that the functional loss of *ndh* genes is the initial stage in the evolution of the plastome of parasitic plants, but the loss has occurred independently multiple times in angiosperms, while they are not found in some parasites. This suggests that the functional loss of *ndh* genes is not essential for the transition from autotroph to parasite. We additionally examined the correlation between gene content and type of parasitism (obligate/facultative and stem/root parasites) of all hemiparasitic species in which cp genomes have been reported to date. Correlation was not found in any types of parasitism.

## Introduction

Parasitism in plants is the result of the transition from autotrophy to heterotrophy, involving a decrease or loss of the ability to photosynthesize, and absorption of the host’s water and nutrients through a haustorium, a specialized structure used to invade hosts [1]. Parasitism in angiosperms is considered to have at least 12 independent origins, and most species are included in the family Orobanchaceae and the order Santalales. Among the 12 recognized lineages of parasitic plants, ca. 4260 species belong to one of two lineages (Orobanchaceae and Santalales), and ca. 220 other species belong to one of 10 lineages (Apodanthaceae, *Cassytha*, *Cuscuta*, Cynomoriaceae, Cytinaceae, Hydnoraceae, Krameriaceae, Lennoaceae, Mitrastemonaceae, and Rafflesiaceae) [2–4]. Depending on photosynthetic ability, parasitic plants are divided into two types: photosynthetic parasites (hemiparasites) and nonphotosynthetic parasites (holoparasites). Hemiparasitic plants possess varying rates of photosynthetic ability, while holoparasitic plants obtain all nutrients from their host and show complete loss of photosynthetic ability [5]. Among the 12 lineages of parasitic plants, hemiparasites are found in *Cassytha*, *Cuscuta*, Krameriaceae, Orobanchaceae, and Santalales. Hemiparasitic Santalales is composed of obligate and facultative parasites that are distinguished by the essential or nonessential role of a host in the parasite’s full life cycle [2, 3].

The transition from autotrophy to heterotrophy in plants leads to pseudogenization and loss of plastid genes associated with photosynthesis. It also leads to reduction and change in the highly conserved plastome, which consists of two copies of large inverted repeats (IRs) that separate the large and small single copy regions (LSC and SSC) [2, 6–12]. The rate of chloroplast genome degradation in parasitic plants varies with the level of photosynthesis [13]. The complete cp genomes of hemiparasitic plants deposited in GenBank to date (https://www.ncbi.nlm.nih.gov/genome, last accessed May 28, 2017) are slightly or not reduced in size compared to typical cp genomes in angiosperms (120–170 kb) [14–16]. In holoparasitic plants, reductions in cp genome size are typically observed [7, 9, 16–21], and in several species, dramatically reduced cp genome sizes are found [22–24]. Even the possible loss of the entire cp genome has been reported in *Rafflesia* [25]. Along with a reduction in cp genome size, the conserved quadripartite genome structure has been perturbed in some holoparasitic species [16, 22, 23]. Among the 13 completed cp genomes of hemiparasitic plants, two species, *Cassytha filiformis* and *Striga hermonthica*, have lost the typical quadripartite structure [9, 26]. Among the 120–130 genes that are common in typical angiosperm cp genomes [14], pseudogenization and loss of *ndh* genes are considered the first steps of gene content change in the evolution of heterotrophic plants [13, 16, 27]. Ndh-specific degradations are commonly found in the currently completed cp genomes of hemiparasitic plants, except for *Triphysaria versicolor*, which is a facultative parasite in Orobanchaceae [9, 15, 16, 26, 28–30]. Unlike hemiparasitic plants, the cp genomes of holoparasitic plants cover the full spectrum of degradation in gene content [23]. In a recent study describing a mechanistic model of plastome degradation and accelerated evolutionary rates, Wicke et al. (2016) suggest that the transition from autotrophy to obligate parasitism relaxes functional constraints on plastid genes in a stepwise manner.

Santalales, which is one of the largest orders in angiosperms, consists of 20 families, ca. 179 genera, and 2460 species [3, 4]. It consists of a relatively small number of autotrophic species and a large number of parasitic species, mostly hemiparasites, which are root or aerial (stem) parasites [31, 32]. The species of Santalales are distributed worldwide, but are found mainly in tropical and subtropical regions. Recent studies of the Santalales have mainly focused on phylogenetic relationships in angiosperms [33–35] and on the phylogenetic classification of Santalales [4, 31, 32, 36–38]. To date, the complete cp genomes of eight Santalales species (*Champereia manillana*, *Osyris alba*, *Schoepfia jasminodora*, *Taxillus chinensis*, *Taxillus sutchuenensis*, *Viscum album*, *Viscum crassulae*, and *Viscum minimum*) belonging to five families (Loranthaceae, Opiliaceae, Santalaceae, Schoepfiaceae, and Viscaceae) have been sequenced [15, 29, 30, 39]. These eight cp genomes have a typical structure, which is highly conserved in most angiosperms and is composed of a single circular DNA molecule with a quadripartite structure including an LSC, SSC, and two IRs. These cp genome sizes range from ca. 119 (*Schoepfia jasminodora*) to 147 (*Champereia manillana*) kb.

In this study, we first report the complete cp genome of *Dendrotrophe varians* in the family Amphorogynaceae. Additionally, from the family Loranthaceae, we report the complete cp genome of *Helixanthera parasitica* of the subtribe Dendrophthoinae and the cp genome of *Macrosolen cochinchinensis* of the tribe Elytrantheae [38]. We describe general characteristics of the three cp genomes based on their structural features such as genome size, gene content, and gene order. To better understand plastome evolution in hemiparasitic Santalales, we compare these three cp genomes with eight other Santalales cp genomes reported and reconfirm their phylogenetic positions. Finally, we investigate pseudogenization and gene loss in parasitic plant cp genomes, with particular focus on hemiparasitic plants.

## Materials and methods

### Plant DNA extraction and cp genome sequencing

*Dendrotrophe varians* was collected from Phnum Bokor National Park, Kampot prov. in Cambodia. *Helixanthera parasitica* and *Macrosolen cochinchinensis* were collected from Phuoc Hoa comm., Bac Ai distr., Ninh Thuan prov. and Sa Son comm., Sa Thay distr., Kon Tum prov. in Vietnam, respectively. A voucher specimen of *Dendrotrophe varians* (CB-2630) was deposited in the Herbarium of Hallym University (HHU) and voucher specimens of *Helixanthera parasitica* (VK4005) and *Macrosolen cochinchinensis* (VK2608) were deposited in the Herbarium of the Institute of Ecology and Biological resources, Hanoi, Vietnam (HN). All three species are not endangered or protected species. Whole genomic DNA was isolated from fresh leaf tissue using the CTAB method [40] or a DNeasy Plant Mini Kit (Qiagen, USA) following the manufacture’s protocols. Extracted DNA was quantified with a NanoDrop spectrophotometer. Raw sequence reads were generated using the Illumina MiSeq Sequencing System (http://www.illumina.com). To extract chloroplast DNA contigs, the raw data were filtered by BLAST searches (National Center for Biotechnology Information, http://blast.ncbi.nlm.nih.gov/Blast.cgi) and assembled with the complete cp genome sequences of Santalales species (*Champereia manillana* [NC_034931], *Osyris alba* [NC_027960], *Schoepfia jasminodora* [NC_034228], *Taxillus chinensis* [KY996492], *T*. *sutchuenensis* [KY996493], *Viscum album* [NC_029039], *Viscum crassulae* [NC_027959], *and Viscum minimum* [NC_027829]), which are deposited in GenBank (https://www.ncbi.nlm.nih.gov/genome, last accessed Nov. 09, 2017), as references using Geneious version 9.1.5 (Biomatters, https://www.geneious.com/). The filtered contigs were assembled de novo, and assembled contigs were used as references. To fill gaps between contigs and to confirm the raw data, direct sequencing of polymerase chain reaction (PCR) products was performed using primers designed from contig ends. To confirm SSC/IR and LSC/IR junctions, repetitive sequences were analyzed using REPuter [41], and a PCR-based survey was also conducted at breakpoints. Additionally, several regions with more than 20 nucleotides of poly A and T were further amplified with PCR to check assembly error of raw contigs from MiSeq sequencing. The PCR products were purified using an AccuPrep PCR Purification Kit (BIONEER) following the manufacturer’s protocols. The PCR products were then sequenced in both directions using the same primers as those used for PCR amplification. Primer information is presented in S1 Table. DNA fragment assembly and sequence editing were performed with Geneious version 9.1.5 (Biomatters, https://www.geneious.com/). All sequences were deposited in GenBank (http://www.ncbi.nlm.nih.gov/genbank/).

### Gene annotation and sequence analysis

BLAST and DOGMA [42] were used to annotate coding genes, and additional annotation was performed in Geneious using reference sequences of Santalales species. The exact positions of the start and stop codons were confirmed, and translation was generated by BioEdit 7.2.5 [43]. The locations of RNAs were predicted by tRNAscan-SE 2.0 [44]. After gene annotation was complete, circular and linear gene maps were drawn by OGDraw 1.2 [45, 46]. Mauve 2.3.1 [47] and Geneious version 9.1.5 (Biomatters, https://www.geneious.com/) were used to compare structural differences among multiple Santalales cp genomes.

### Phylogenetic analysis

To confirm the exact topology among Santalales species, we performed phylogenetic analysis using eight complete cp genome sequences of Santalales available in GenBank, with the three complete cp genome sequences of *Cornus controbersa* [NC_030260], *Diospyros lotus* [NC_030786], and *Fagopyrum esculentum* subsp. *ancestrale* [NC_010776] as outgroup taxa. Fifty-eight protein-coding sequences that are shared among the 14 representative taxa were used for the analysis. Each gene was aligned using MUSCLE 3.5 [48], and then the aligned genes were concatenated into a dataset. Maximum likelihood (ML) analysis was used to construct a phylogenetic tree as performed in RAxML 8.2.7 [49], and it was implemented in Geneious version 9.1.5. The analysis was performed using the GTR+GAMMA+I model generated by Modeltest [50], and clade support was evaluated by 1000 bootstrap replications. Bayesian phylogenetic analysis was performed in MrBayes 3.2.6 [51]. In the Bayesian phylogenetic analysis, the Markov Chain Monte Carlo (MCMC) algorithm approach was used under the parameter settings of chain length = 1,100,000, heated chains = 4, heated chain temperature = 0.2, subsampling frequency = 200, burn in length = 100,000, and random seed = 18,770.

## Results

### The cp genome structure of three newly sequenced species

From genomic DNAs, 7,245,848 raw reads of *Dendrotrophe varians*, 8,931,796 raw reads of *Helixanthera parasitica*, and 9,108,812 raw reads of *Macrosolen cochinchinensis* were produced. The complete cp genomes of *Dendrotrophe varians* (140,666 bp), *Helixanthera parasitica* (124,881 bp), and *Macrosolen cochinchinensis* (122,986 bp) were determined (S1 Fig). The cp genome sizes are in the range of the previously reported Santalales cp genomes. In Loranthaceae, the cp genomes of *Helixanthera parasitica* and *Macrosolen cochinchinensis* are similar in size to the previously reported cp genomes of Loranthaceae species, *Taxillus chinensis* (121,363 bp) and *Taxillus sutchuenensis* (122,562 bp). The eight publicly available cp genomes of hemiparasitic Santalales have a typical organization, divided into four parts: two IRs, an LSC, and an SSC. The cp genomes of the three newly sequenced species are collinear to those of most Santalales species except *Osyris alba* and *Viscum minimum*, which have large inversions. In the cp genome of *Dendrotrophe varians*, there are a total of 101 genes, including 67 protein-coding genes, 4 ribosomal RNA (rRNA) genes, and 30 transfer RNA (tRNA) genes. Sixteen genes (5 protein-coding genes, 4 rRNA genes, and 7 tRNA genes) have been duplicated in IRs. One pseudogene (*ψinfA*) was detected and has a premature stop codon. Two partial sequences of *ndhB* genes were detected in IRs. One truncated gene of *rps19* is located at the LSC-IRA junction. The *Helixanthera parasitica* and *Macrosolen cochinchinensis* cp genomes have an identical number of genes. In each genome, there are a total of 92 genes including 63 protein-coding genes, 4 rRNA genes, and 25 tRNA genes. Thirteen genes (4 protein-coding genes, 4 rRNA genes, and 5 tRNA genes) were duplicated in IRs. The *infA* gene is lost in the *Helixanthera parasitica* cp genome, and the *rpl36* gene is lost in the *Macrosolen cochinchinensis* cp genome. Two pseudogenes, *ψinfA* gene in the *Helixanthera parasitica* cp genome and *ψrpl16* gene in the *Macrosolen cochinchinensis* cp genome, were detected. Both pseudogenes have premature stop codons. The two cp genomes have the same gene segments in IRs and truncated genes at the IR junctions. The partial sequences of *ndhB*, *trnA-UGC*, and *trnI-GAU* genes were detected in IRs. Two truncated genes of *rpl2* and *ycf1* are located at the LSC-IRA and SSC-IRB junctions, respectively.

### General features of Santalales cp genomes

We compared general features of 11 Santalales cp genomes including three newly sequenced cp genomes (Table 1). The largest cp genome is *Champereia manillana* (147,461 bp) and the smallest cp genome is *Schoepfia jasminodora* (118,743 bp) which is reduced by 28,718 bp (19.5%) compared to the *Champereia manillana* cp genome. The reduction is due to the contraction of protein-coding regions. Among the LSC, SSC, and IR regions, many contractions in the IR regions are responsible for the reduction of the *Schoepfia jasminodora* cp genome. We newly annotated eight cp genomes, which were previously deposited in GenBank, and compared all 11 cp genomes with one another to get the most accurate genome annotation. Inaccurate annotation of genes in the previously deposited genomes was due to typing errors in tRNA genes, incorrect prediction of gene location, and miscounted genes because of gene duplication. The Santalales cp genomes have a total of 91–101 genes including duplicated genes in the IRs. These genes consist of 63–69 protein-coding genes, 23–30 tRNA genes, and 4 rRNA genes. All annotated genes are listed in S2 Table. Among the three major regions (LSC, SSC, and IRs), the IRs have the highest GC content, with average of 43.3%, and the SSC has the lowest GC content, with an average of 26.8%. The GC content of the cp genome of *Schoepfia jasminodora* is the highest, and that of *Viscum minimum* is the lowest of all the cp genomes. The *Viscum crassulae* cp genome has the most compact cp genome with a total of 62.1% coding regions.

**Table 1:** Comparison of cp DNA features among 11 Santalales species.

Features		*Viscum minimum*	*Viscum crassulae*	*Viscum* *album*	*Dendrotrophe varians*	*Osyris* *alba*	*Champereia manillana*	*Taxillus sutchuenensis*	*Taxillus chinensis*	*Helixanthera parasitica*	*Macrosolen cochinchinensis*	*Schoepfia jasminodora*
Size (bp)												
Total		131,016	126,064	128,921	140,666	147,253	147,461	122,562	121,363	124,881	122,986	118,743
	Protein-coding genes	66,691	66,562	64,231	64,624	66,500	66,254	63,593	63,722	64,010	64,100	58,362
	tRNA genes	2,735	2,662	2,736	2,792	2,656	2,792	2,139	2,141	2,287	2,287	2,559
	rRNA genes	9,034	9,044	9,050	9,068	9,044	9,049	9,072	9,072	9,062	9,052	9,050
	introns	15,314	15,278	17,161	16,434	16,648	10,052	6,991	6,858	9,091	8,694	13,971
	IGSs	37,242	32,518	35,743	47,748	52,405	59,314	40,767	39,570	40,431	38,853	34,801
	LSC	75,814	73,226	73,893	81,684	84,601	83,505	70,630	70,357	73,043	69,018	84,168
	SSC	9,014	8,628	8,632	10,870	13,972	7,806	6,102	6,082	6,334	6,144	9,763
	one IR	23,094	22,105	23,198	24,056	24,340	28,075	22,915	22,462	22,752	23,912	12,406
Number of genes												
Total		99	98	96	101	101	101	91	91	92	92	101
	Protein-coding genes	66	66	64	67	67	67	64	64	63	63	69
	tRNA genes	29	28	28	30	30	30	23	23	25	25	28
	rRNA genes	4	4	4	4	4	4	4	4	4	4	4
	Genes with introns	15	14	14	16	16	16	8	8	10	10	14
% GC content												
Total		36.2	36.4	36.4	37.8	37.7	37.4	37.3	37.3	36.5	36.6	38.1
	LSC	33.3	33.6	33.5	35.5	35.6	35.3	34.7	34.7	33.8	33.6	36.1
	SSC	24.2	24.0	24.8	29.7	31.2	27.9	26.2	26.2	25.5	24.4	30.7
	IRs	43.2	43.4	43.2	43.7	43.1	41.9	42.8	43.0	42.3	42.2	47.9
% Coding regions												
Total		59.8	62.1	59.1	54.4	53.5	53.0	61.1	61.8	60.3	61.3	59.0
	Protein-coding genes	50.9	52.8	49.8	45.9	45.2	44.9	51.9	52.5	51.3	52.1	49.2
	tRNA genes	2.1	2.1	2.1	2.0	1.8	1.9	1.7	1.8	1.8	1.9	2.2
	rRNA genes	6.9	7.2	7.0	6.4	6.1	6.1	7.4	7.5	7.3	7.4	7.6

### Loss of genes throughout Santalales phylogeny

**Fig 1:**
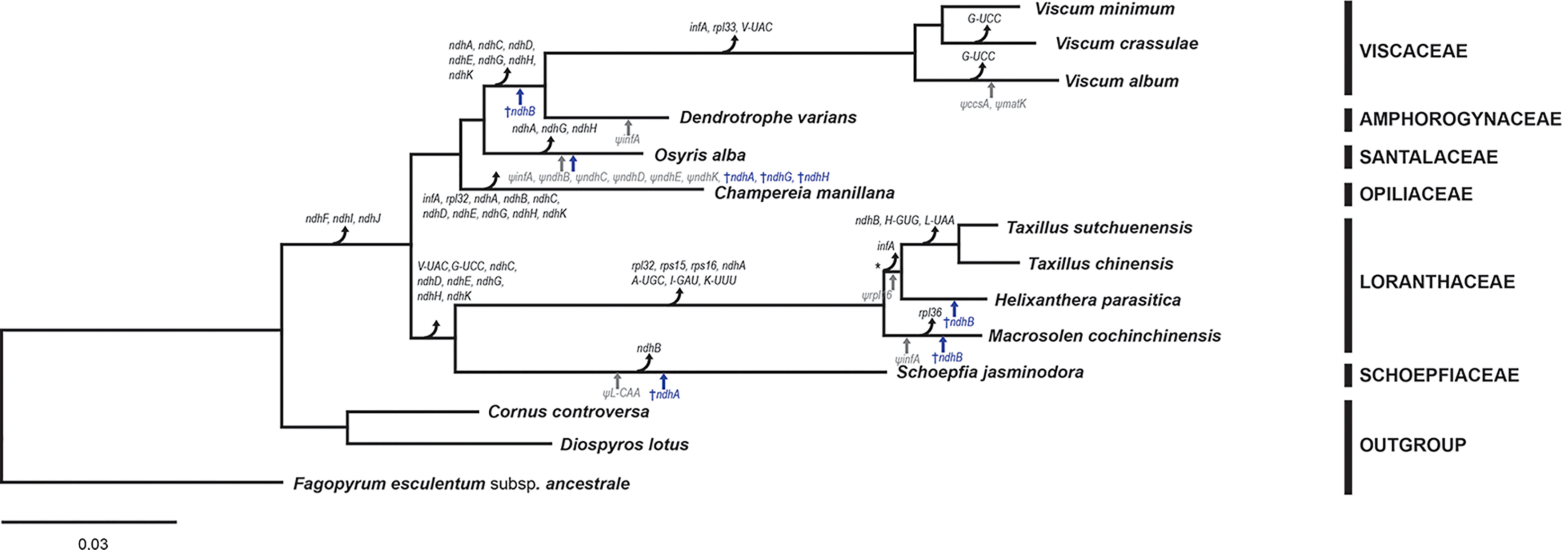
Maximum likelihood tree resulting from the 58 concatenated protein-coding genes common to 14 representative taxa. The BI tree had identical topology with the ML tree. The GTR+I+G model was selected based on Modeltest. The bootstrap support values of the ML tree and the posterior probabilities of the BI tree at all nodes are 100% and 1.00, respectively, except for one node marked by an asterisk, which has a value of 99% and probability of 1.00. *ψ*, pseudogene; †, partial gene sequence.

To confirm the phylogenetic relationships among Santalales species, a phylogenetic tree was constructed using 58 concatenated protein-coding genes common to all taxa (Fig 1). As in previous studies of global angiosperm phylogeny [34, 35], monophyly of the Santalales was well supported, with a bootstrap value of 100%. All branches were supported by 100% bootstrap values, except for the Loranthaceae clade, which had a 99% bootstrap value. *Dendrotrophe varians* of the Amphorogynaceae was sister to the Viscaceae, and *Schoepfia jasminodora* of the Schoepfiaceae was sister to the Loranthaceae. A clade made up of Loranthaceae and Schoepfiaceae was sister to a clade consisting of Viscaceae, Amphorogynaceae, Santalaceae, and Opiliaceae. The functional loss of all *ndh* genes occurred in all 11 Santalales species. Depending on the species, the *ndh* genes were completely lost, or several of the *ndh* genes have recognized sequences as pseudogenes or partial sequence of genes. Six genes have been lost (*rpl32*, *rps15*, *rps16*, *trnA-UGC*, *trnI-GAU*, and *trnK-UUU*) in the Loranthaceae. Parallel gene loss or pseudogenization of *infA* was detected in some branches. The gene *trnG-UCC* was lost independently in *Viscum crassulae*, *Viscum album*, and the clade of Loranthaceae and Schoepfiaceae. Ten pseudogenes were detected in Santalales cp genomes. The *ψccsA* and *ψmatK* genes are in the *Viscum album* cp genome. The *ψinfA* genes are in the *Dendrotrophe varians*, *Macrosolen cochinchinensis*, and *Osyris alba* cp genomes. The *ψndhB*, *ψndhC*, *ψndhD*, *ψndhE*, and *ψndhK* genes are in *Osyris alba* cp genomes. The *ψrpl16* genes are in two *Taxillus* species and *Helixanthera parasitica* cp genomes. The *ψtrnL-CAA* is in the *Schoepfia jasminodora* cp genome.

### Evolutionary dynamics of inverted repeat regions

**Fig 2:**
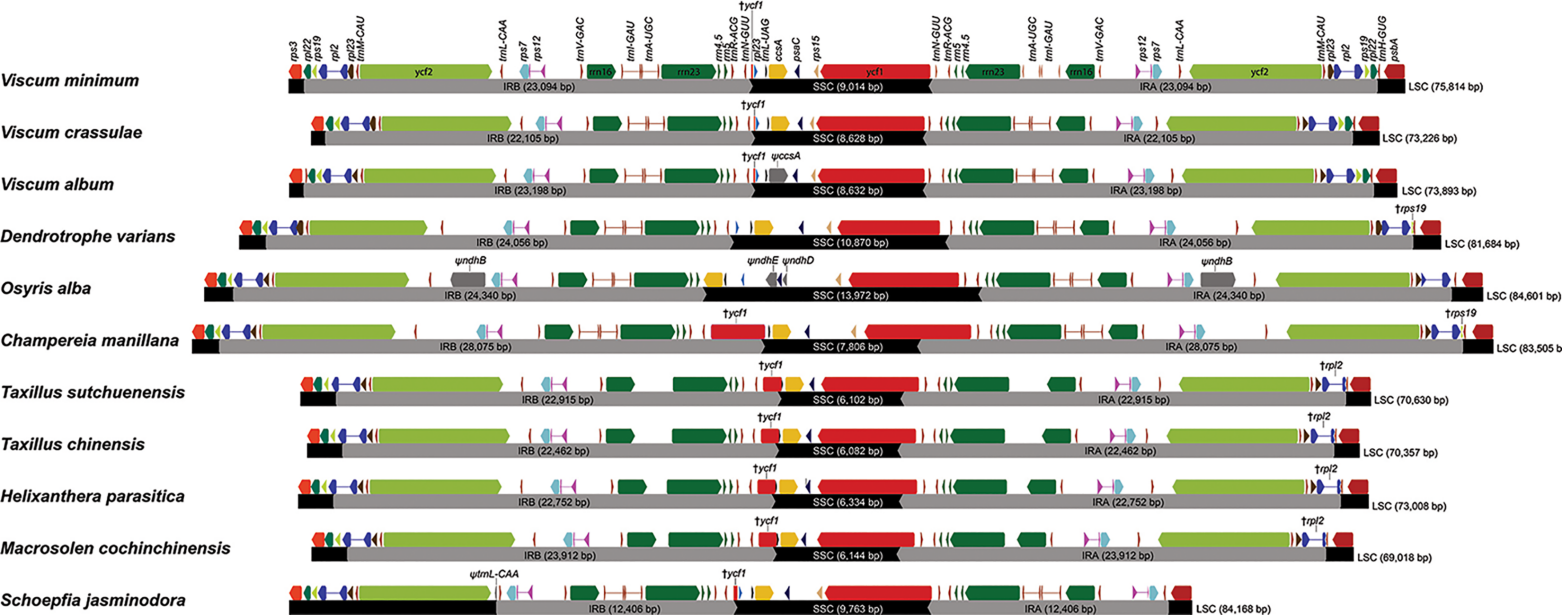
Comparison of the SSC-IR and LSC-IR boundary regions across 11 Santalales species.

We compared IR/LSC and IR/SSC junctions in 11 Santalales cp genomes (Fig 2). Santalales species show various sizes of IRs, caused by the evolutionary dynamics of expansion and contraction. They commonly have six duplicated genes (*rrn16*, *rrn23*, *rrm4*.*5*, *rrn5*, *rps7*, and *trnV-GAC*) and two exons of *rps12* in their IRs. All cp genomes except *Dendrotrophe varians* and *Osyris alba* have truncated *ycf1* genes, which are produced by the presence of IR/SSC junctions within the *ycf1* genes. The gene number and order of the IRs in Loranthaceae cp genomes are identical. The IR/LSC junctions of all cp genomes of Loranthaceae species are in exon 2 of *rpl2* genes. In the Viscaceae, three *Viscum* have similar gene numbers and order of genes. The *Viscum album* cp genome has the most expanded IRs toward the LSC. After expanding from the IRB to the LSC up to the point between the *rps3* and *rpl22* genes, the IRA extends up to the *trnH-GUG* gene. The IRs of the cp genomes of *Viscum crassulae* and *Viscum minimum* do not include the *trnH-GUG* genes. The *Champereia manillana* cp genome, which has the largest IRs (28,075 bp), also has the largest truncated *ycf1* gene (2,777 bp) at the junction of IRB/SSC. The *Schoepfia jasminodora* cp genome has the most highly contracted IRs (12,406 bp) and is the only cp genome in which the IRs do not include duplicated *ycf2* genes.

### Gene content of hemiparasitic plants

**Fig 3:**
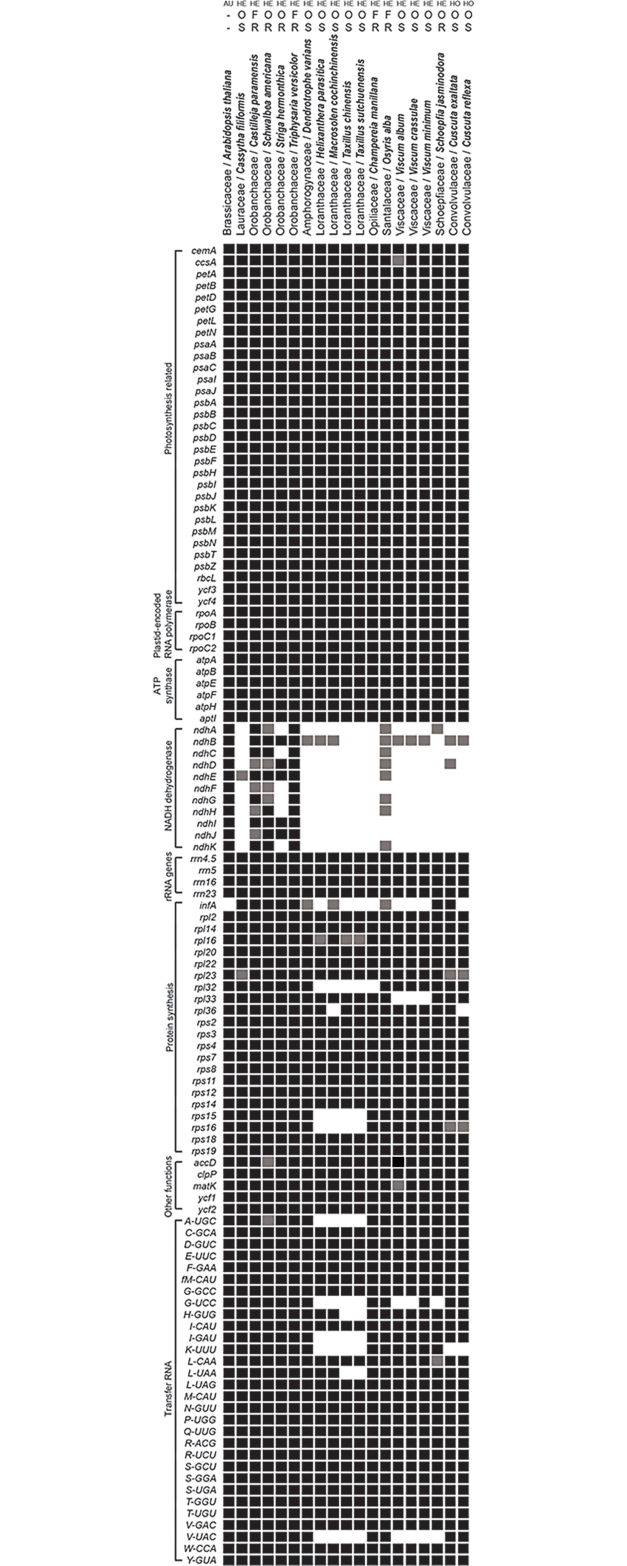
Comparison of the cp genome gene content of 16 hemiparasitic plants published to date with one autotrophic plant and two *Cuscuta* species, based on Funk, H. T., et al. (2007), McNeal, J. R., et al. (2007), Wicke, S., et al. (2013), Petersen, G., et al. (2015), Rossetto, M., et al. (2015), Fan, W., et al. (2016), Su, H. -J. and J. -M. Hu (2016), Wicke, S., et al. (2016), Li, Y., et al. (2017) and Yang, G. -S., et al. (2017). In the case of *Striga hemonthica*, pseudogene presence was not confirmed in a previous paper. Black/gray/white boxes indicate each gene present and are considered functional/pseudogene or fragment of gene present/gene absent. AU, autotroph; HE, hemiparasite; HO, holoparasite; O, obligate parasite; F, facultative parasite; S, stem parasite; R, root parasite.

Gene content of complete cp genomes of all hemiparasitic plants published to date, including one autotrophic plant *Arabidopsis thaliana* and two *Cuscuta* species that are considered ‘photosynthetic holoparasites’ or hemiparasites, are summarized in Fig 3. Among all hemiparasitic plants deposited in GenBank, the cp genomes of *Bartsia inaequalis* and *Pedicularis ishidoyana* were excluded due to being incomplete sequences. All genes were categorized into 8 different functional groups. Among the eight functional gene groups, the NADH dehydrogenase gene group is highly variable, with intactness, pseudogenization, or complete loss of genes, while the rRNA gene and ATP synthase groups are intact in all species. All hemiparasitic species except the species of Orobanchaceae share the functional loss of whole *ndh* genes. In the Santalales, the cp genome of *Osyris alba* has several *ndh* pseudogenes (*ndhB*, *ndhC*, *ndhD*, *ndhE*, and *ndhK*) [28, 29], but the cp genomes of other species lost all *ndh* genes completely or have only a partial sequence of *ndhA* or *ndhB* gene. The *Dendrotrophe varians* cp genome has only the exon 2 fragment of the *ndhB* gene, but the *Helixanthera parasitica* and *Macrosolen cochinchinensis* cp genomes have only the exon 1 fragment. Three *Viscum* cp genomes are composed of exon 1 and exon 2 fragments of *ndhB* gene.

We categorized each species as autotroph/hemiparasite/holoparasite, obligate/facultative parasite, and stem/root parasite according to ecological and morphological characteristics. The one facultative root parasite, *Triphysaria versicolor* of the Orobanchaceae, has all *ndh* genes intact, while the other facultative and root parasites, *Champereia manillana* and *Osyris alba* of the Santalales, have no intact *ndh* genes. The obligate and root parasites functionally lost all *ndh* genes or have several intact *ndh* genes. All obligate and stem parasites show the functional loss of all *ndh* genes, but they are different in the degree of gene degradation. In the case of other genes, there is no correlation of parasitic types (obligate/facultative parasite and stem/root parasite) and degree of gene degradation.

## Discussion

In the order Santalales, which is a large order including most hemiparasites, more studies on its molecular phylogeny and genomics are needed [31, 32, 38]. The cp genomes of hemiparasitic plants have smaller genetic changes than those of holoparasitic plants, but studies of hemiparasites are important for understanding the evolutionary transition from autotrophs to parasites. In previous molecular phylogenetic studies, the evolutionary relationships of Santalales species have been confirmed by plastid genes (*accD*, *matK*, *rbcL*, and *trnL-F*), nuclear genes (SSU rDNA, LSU rDNA, and RPB2), and a mitochondrial gene (*matR*) [4, 31, 32, 36]. In this study, most protein-coding genes in the cp genomes of Santalales species were used to confirm the phylogeny of 11 Santalales species belonging to six families. The topologies of the six families are the same as the topologies in the recently revised classification system based on molecular and morphological data [38]. We confirmed that *Dendrotrophe varians* of Amphorogynaceae is sister to the three *Viscum* species of Viscaceae, and that *Helixanthera parasitica* and *Macrosolen cochinchinensis* form a clade with two *Taxillus* species to make up the family Loranthaceae (Fig 1).

The 11 Santalales species have a slightly reduced number of genes in their cp genomes compared to typical angiosperm cp genomes (Table 1) [14]. Common losses of some genes are related to phylogenetic relationships. Some losses of genes in each clade, such as the common losses of the *rpl32*, *rps15*, *rps16*, *A-UGC*, *I-GAU*, and *K-UUU* genes in the Loranthaceae, are considered important characteristics of families, genera, or species (Figs 1 and 3). However, the genes lost in Santalales cp genomes are also lost in other angiosperm lineages and represent phylogenetically independent losses [52]. The parallel loss and pseudogenization of *ndh* genes and various fragments that are composed of exon 1, exon 2, or both exons of the *ndhB* gene were detected independently in the phylogeny of Santalales. The *rpl32*, *trnG-UCC*, and *infA* genes were also independently lost or pseudogenized. The gene losses in hemiparasitic Santalales cp genomes are not as extreme as those in holoparasitic plant cp genomes, which have few or no genes [22, 23, 25]. These gradual losses enhanced the understanding about evolutionary history in hemiparasitic Santalales cp genomes. The families of Santalales have a confusing taxonomic history [38, 53–59]. To further clarify phylogenetic relationships among Santalales families, genera, and species, more morphological and molecular data of taxa belonging to all Santalales families are required. Additionally, the cp genomes of autotrophic Santalales species are needed for more in-depth analysis of the evolutionary transition from autotrophs to parasites.

Generally, closely related species tend to have similar IR boundaries with few changes [60–62]. The cp genomes of Santalales species have similar IR boundaries in that the IRB/LSC junctions are near the *rpl2*, *rps19*, and *rpl22* genes except for the *Viscum album* and *Schoepfia jasminodora* cp genomes (Table 1 and Fig 2). Expansions from the IRB to the LSC up to *rpl2* are common in angiosperms, and expansions up to *rps19* or *rpl22* are often found in vascular plants [60]. The IR/LSC and IR/SSC junctions of all four Loranthaceae species are located at the same positions, which are within exon2 of *rpl2* and *ycf1*. Most angiosperms have 20–22 kb of one IR [63, 64]. The size of one IR in four species of Loranthaceae and *Dendrotrophe varians* of the Amphorogynaceae is slightly bigger than that of most angiosperms. In the Santalales, the cp genome of *Schoepfia jasminodora* has highly contracted IRs (12,406 bp), which are reduced until the IRs do not include the *ycf2* gene (Fig 2). Goulding et al. (1996) suggested two IR expansion models. The first model is a single-strand break and gene conversion leading to a small IR expansion, and the second model is a double-strand break resulting in a larger IR expansion [62]. Among 11 Santalales species, the cp genomes of *Osyris alba* and *Viscum minimum* have large inversions and a general structure consisting of two IRs (IRA and IRB) and two single copy regions (LSC and SSC) [29]. These may be caused by mechanisms such as mediation of duplicated tRNA genes [65, 66], mediation of dispersed short repeats [67–70], and HR (homologous recombination) between more than 200 bp inverted repeats [11]. The two IRs of land plant plastomes typically contain four rRNA genes (*rrn16*, *rrn23*, *rrn4*.*5* and *rrn5*) and five tRNA genes (*trnA-UGC*, *trnI-GAU*, *trnN-GUU*, *trnR-ACG*, and *trnV-GAC*). In the Loranthaceae, the IRs do not include the intron-containing tRNA genes *trnA-UGC* and *trnI-GAU*. Large structural changes in the plastome are evolutionarily and phylogenetically important characteristics in land plants [71]. The Santalales cp genomes also show various large structural changes such as gene deletion, IR expansion, and large inversions. Future phylogenetic studies in the Santalales should analyze additional closely related species and their structural genomic changes.

The *ndh* complex is the only group (among eight functional groups) that has functionally lost all genes in Santalales. Like the cp genomes of previously reported Santalaceae species, *Dendrotrophe varians*, *Helixanthera parasitica*, and *Macrosolen cochinchinensis* cp genomes have functionally lost all *ndh* genes. This supports the idea that the functional loss of *ndh* genes is the initial stage in the transition from autotrophs to parasites [16, 27, 28]. The loss of *ndh* genes has occurred independently multiple times in angiosperms [52]. It is not only found in various parasitic plants with 12 independent origins, but also in several non-parasitic lineages: the Pinaceae, Gnetales, Orchidaceae, etc. The *ndh* complex is considered inessential to the plant cp genome, based on previous studies examining varying degrees of loss and pseudogenization in the *ndh* complex [16, 52, 72].

We categorized the hemiparasitic species as obligate/facultative and stem/root parasites to examine the correlation between parasitic types and gene content of hemiparasitic plants. Unlike an obligate parasite, a facultative parasite can survive without a host, like an autotroph, and is an opportunistic parasite [73]. Its cp genome may be expected to be similar in size and gene number to that of autotrophs. Four facultative hemiparasites, *Castilleja paramensis*, *Champereia manillana*, *Osyris alba*, and *Triphysaria versicolor*, have different degrees of *ndh* gene loss, ranging from containing all genes (*Triphysaria versicolor*) to none (*Champereia manillana* and *Osyris alba*). Additionally, the *Champereia manillana* cp genome lost *infA* and *rpl32* genes, and the *infA* gene of the *Osyris alba* cp genome was pseudogenized. The degrees of gene loss and pseudogenization in obligate hemiparasites also vary. The range of cp genome size of four facultative hemiparasites is from 147,253 to 152,448 bp, which is included in the range for obligate hemiparasites (from 121,363 to 160,910 bp). There is no tendency for gene content or cp genome size to be related with parasitic type of obligate or facultative parasitism. A common change in gene content in stem or root parasites was also not detected. From this, it may be concluded that the degrees of change in cp genome size and gene content of hemiparasitic plants are not correlated with parasitic type.

## Supporting information

S1 Fig Gene maps of *Dendrotrophe varians*, *Helixanthera parasitica*, and *Macrosolen cochinchinensis* cp genomes. Genes drawn inside the circle are transcribed clockwise, while those drawn outside the circle are transcribed counterclockwise. Dark and light gray bars of the inner circle are graphs of GC and AT content, respectively. *ψ*, pseudogene; †, truncated gene at the LSC/IR or SSC/IR junction. The *infA* gene marked by an asterisk is a pseudogene in the *M*. *cochinchinensis* cp genome. The *rpl16* gene marked by two asterisks is a pseudogene in the *H*. *parasitica* cp genome. The *rpl36* gene marked by three asterisks is lost in the *M*. *cochinchinensis* cp genome.
(JPG)

S1 Table Primers used to confirm junction regions of LSC/IR and SSC/IR. (PDF)

S2 Table Gene content of 11 Santalales cp genomes. +, gene present and considered functional; -, gene not present or not functional. *ψ*, pseudogene; †, partial gene sequence including truncation at the LSC/IR or SSC/IR junction. Gray shaded boxes indicate genes in IRs.
(XLSX)
