# Correction: Understanding plastome evolution in Hemiparasitic Santalales: Complete chloroplast genomes of three species, *Dendrotrophe varians*, *Helixanthera parasitica*, and *Macrosolen cochinchinensis*

**DOI:** 10.1371/journal.pone.0205616

**Published:** 2018-10-05

**Authors:** Hye Woo Shin, Nam Sook Lee

The Data Availability statement for this paper is incorrect. The correct statement is: The data underlying this study have been uploaded to GenBank and are available using the following accession numbers: MG808039.1, MF592987.1, and MG808038.1.
