# Supplementary figures and images for: Understanding plastome evolution in Hemiparasitic Santalales: Complete chloroplast genomes of three species, Dendrotrophe varians, Helixanthera parasitica, and Macrosolen cochinchinensis
Source: PLoS One. 2018 Jul 5;13(7):e0200293. doi: 10.1371/journal.pone.0200293 (PMC6033455; doi:10.1371/journal.pone.0200293)

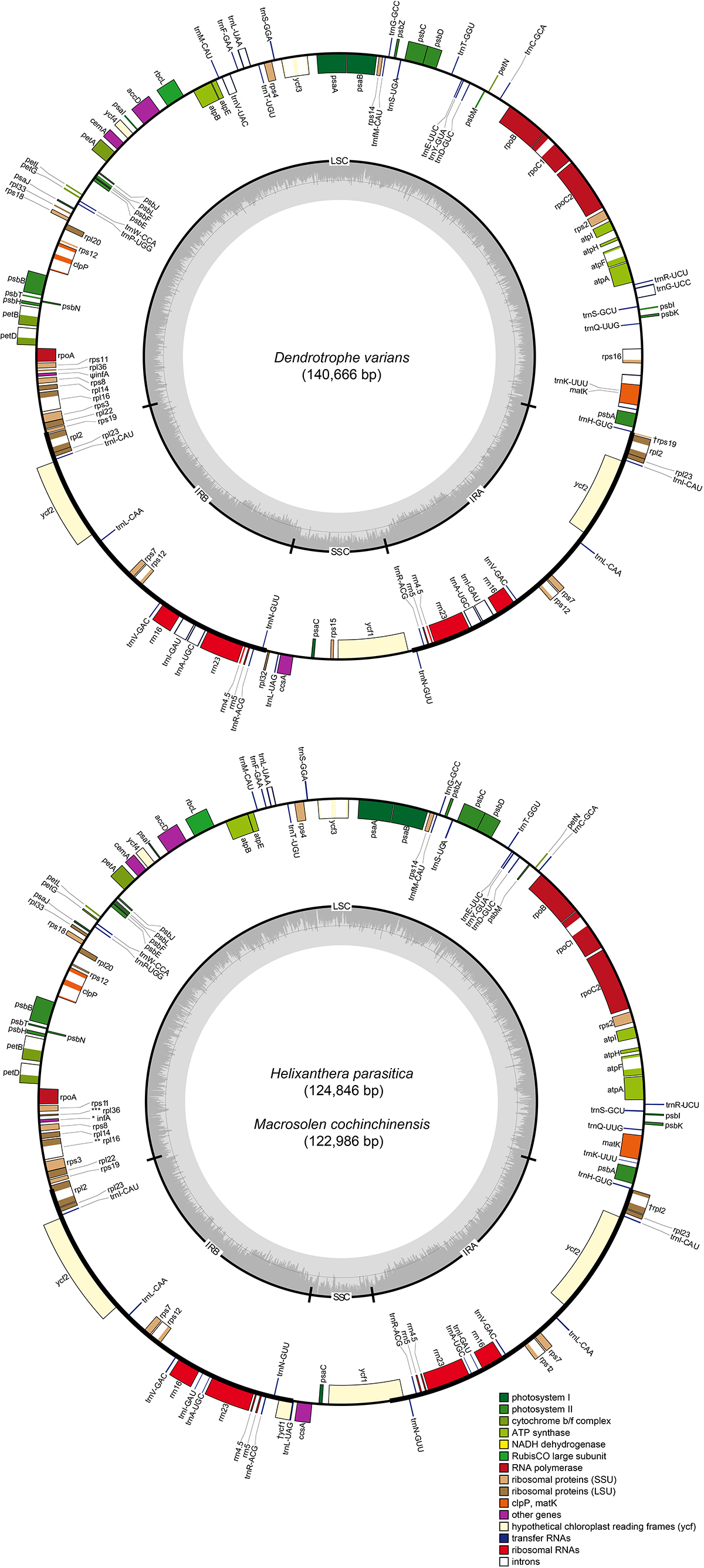

Supplement: S1 Fig — Genes drawn inside the circle are transcribed clockwise, while those drawn outside the circle are transcribed counterclockwise. Dark and light gray bars of the inner circle are graphs of GC and AT content, respectively. ψ, pseudogene; †, truncated gene at the LSC/IR or SSC/IR junction. The infA gene marked by an asterisk is a pseudogene in the M. cochinchinensis cp genome. The rpl16 gene marked by two asterisks is a pseudogene in the H. parasitica cp genome. The rpl36 gene marked by three asterisks is lost in the M. cochinchinensis cp genome. (JPG) [file pone.0200293.s001.jpg]
