# Supplementary material for: Understanding plastome evolution in Hemiparasitic Santalales: Complete chloroplast genomes of three species, Dendrotrophe varians, Helixanthera parasitica, and Macrosolen cochinchinensis
Source: PLoS One. 2018 Jul 5;13(7):e0200293. doi: 10.1371/journal.pone.0200293 (PMC6033455; doi:10.1371/journal.pone.0200293)
Supplement: S1 Table — (PDF) [file pone.0200293.s002.pdf]

S1 Table. Primers used to confirm junction regions of LSC/IR and SSC/IR.

Primer name	Primer sequence (5'-3')	Product size (bp)	Location	Reference
**Lo_IRB/SSC_F**	TTGGTTGGTTAATTGGTCACA	808-819 bp	IR (*ycf1*)	four cp genomes of Loranthaceae species
**Lo_IRB/SSC_R**	AATGGAGAAACCCCATGAAA		SSC (*ccsA*)	
**Lo_SSC/IRA_F**	AATTCTTTGTCTAGARCTTCAATTCT	719-737 bp	SSC (*ycf1*)	
**Lo_SSC/IRA_R**	TTGGTTGGTTAATTGGTCACA		IR (*ycf1*)	
**Lo_LSC/IRB_F**	TGGTAGATGCTCGTGACCAA	584-605 bp	LSC (*rps19*)	
**Lo_LSC/IRB_R**	GGCACGGCCATACATAACAT		IR (*rpl2*)	
**Lo_IRA/LSC_F**	GGCACGGCCATACATAACAT	432-546 bp	IR (*rpl2*)	
**Lo_IRA/LSC_R**	TGCATGAACGTAATGCTCATAAT		LSC (*psbA*)	
**De_IRB/SSC_F**	AGAATCGCGCAGAACAGAAT	619 bp	IR (*trnR-ACG/trnN-GUU*)	*Dendrotrophe varians* cp genome
**De_IRB/SSC_R**	CCCAGTATACCCTCCTTTTCC		SSC (*rpl32*)	
**De_SSC/IRA_F**	CCTAATGCTAGGATGCAGAGG	737 bp	SSC (*ycf1*)	
**De_SSC/IRA_R**	AGAATCGCGCAGAACAGAAT		IR (*trnR-ACG/trnN-GUU*)	
**De_LSC/IRB_F**	TTTGGGTTCCCTCCTTTCTT	656 bp	LSC (*rpl22*)	
**De_LSC/IRB_R**	CCAAAAACTGCTCAGCAACA		IR (*rpl2*)	
**De_IRA/LSC_F**	CCAAAAACTGCTCAGCAACA	775 bp	IR (*rpl2*)	
**De_IRA/LSC_R**	TGTTGAAGTTCCGTCCACAA		LSC (*psbA*)	
